# Ocular Adnexal Manifestations of Sporotrichosis: A Report of Two Cases

**DOI:** 10.7759/cureus.59939

**Published:** 2024-05-08

**Authors:** Leroy Tan, Nurulhuda Ariffin, Francesca Martina M Vendargon, Safinaz Mohd Khialdin

**Affiliations:** 1 Department of Ophthalmology, Universiti Kebangsaan Malaysia Medical Centre, Kuala Lumpur, MYS; 2 Department of Ophthalmology, Hospital Sultanah Aminah, Johor Bahru, MYS; 3 Department of Ophthalmology, Hospital Universiti Kebangsaan Malaysia, Kuala Lumpur, MYS

**Keywords:** ocular adnexal sporotrichosis, conjunctival granuloma, parinaud's oculoglandular syndrome, sporothrix schenckii species, ocular sporotrichosis

## Abstract

Sporotrichosis is a subcutaneous mycotic infection, caused by the dimorphic fungi *Sporothrix schenckii*. Ocular sporotrichosis has both intraocular and adnexal forms. We describe two cases of sporotrichosis involving the conjunctiva of two healthy individuals after inoculation by their pet cats, with complete resolution of lesions after antifungal treatment.

## Introduction

Sporotrichosis is a subcutaneous mycotic infection caused by the dimorphic fungus *Sporothrix schenckii*. Ocular sporotrichosis has been reported to have both intraocular and adnexal forms. We present two cases of adnexal ocular sporotrichosis in immunocompetent adults successfully treated with oral antifungals.

## Case presentation

Case 1

A 62-year-old healthy gentleman presented with a two-week history of painless left eye redness with minimal purulent discharge and was initially treated with topical chloramphenicol for bacterial conjunctivitis.

**Figure 1:**
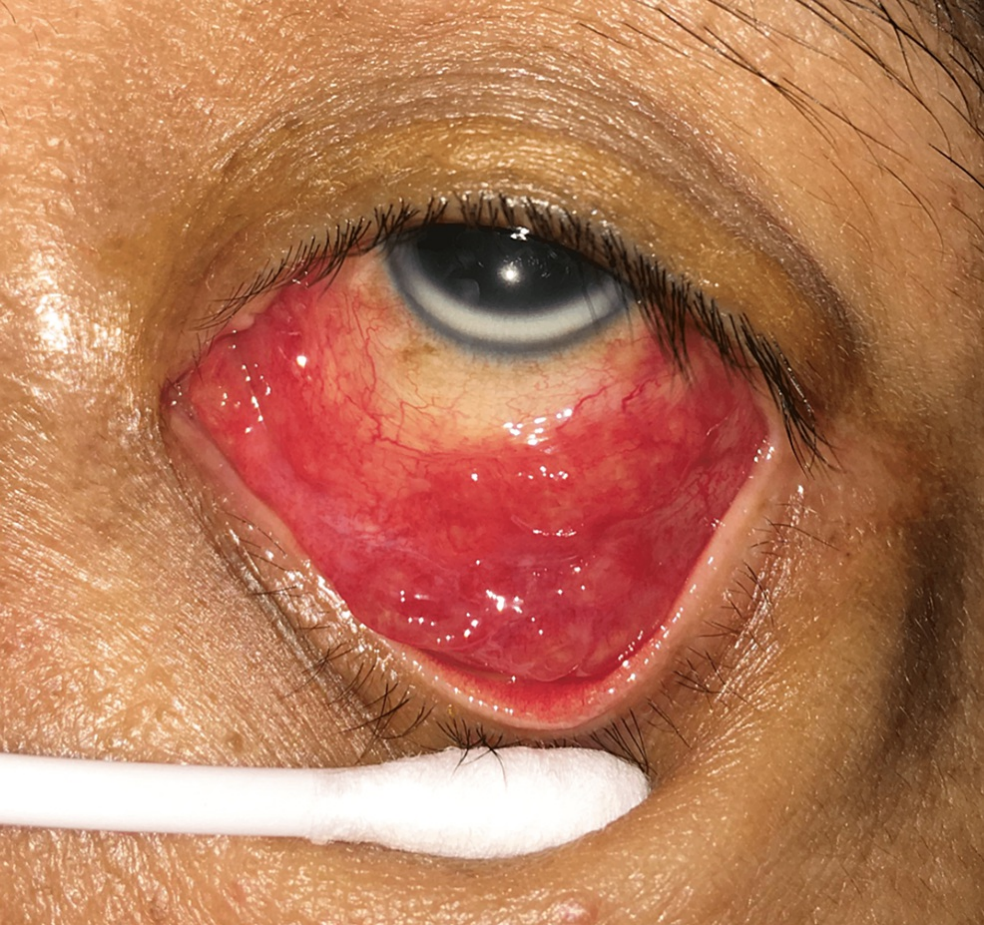
The appearance of multiple and confluent conjunctival granulomata of the lower eyelid conjunctiva. A similar appearance was seen in the upper eyelid conjunctiva. Pictures were taken and published with consent from the patient.

**Figure 2:**
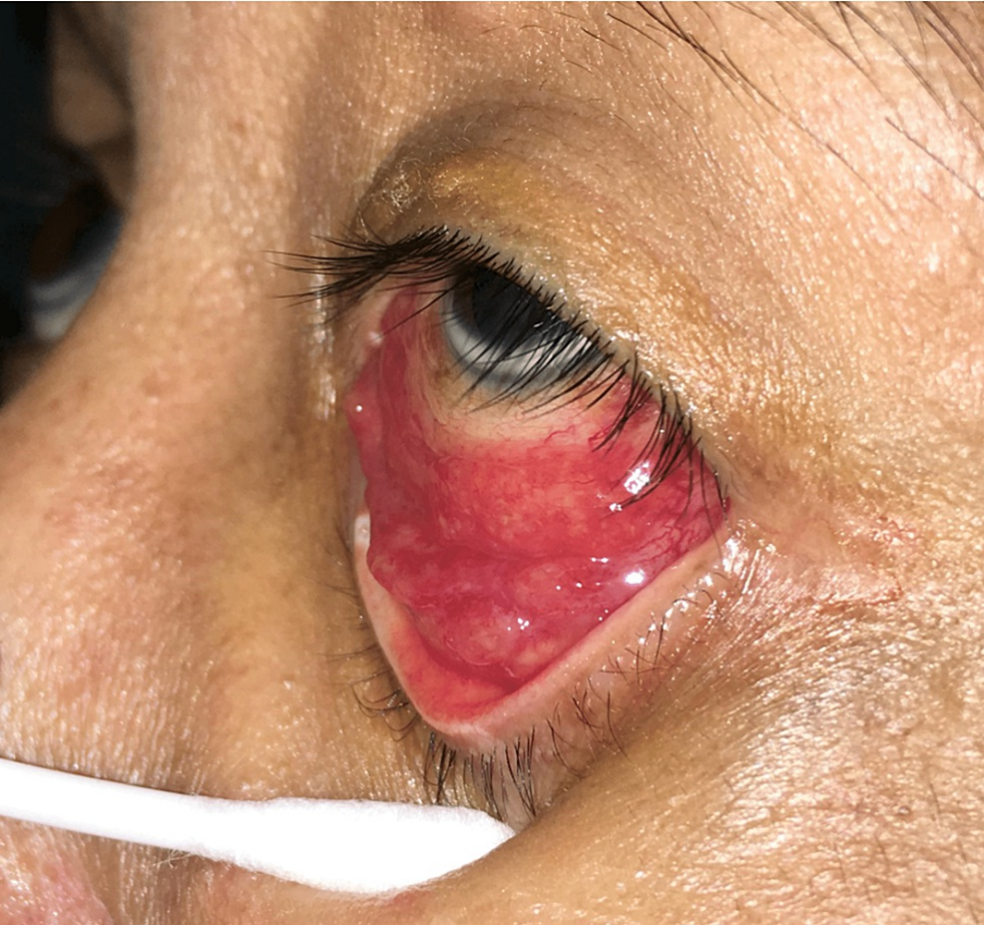
Image showing the same lesion from a lateral view.

He returned a week later with persistent left eye redness and discharge. Moreover, the left eye had become swollen. Examination revealed left conjunctival granulomata involving both tarsal and bulbar conjunctivae. Figures 1, 2 show the appearance of multiple and confluent conjunctival granulomata of the lower eyelid conjunctiva. A similar appearance was seen in the upper eyelid conjunctiva.

Visual acuity was not affected, and there were no abnormal ocular findings. There was neither lymphadenopathy nor a remarkable systemic examination. The patient kept a pet cat, but there were no incidences of cat scratches. Further history-taking revealed that his cat was on treatment for a skin lesion called "Sporo."

A presumed diagnosis of oculoglandular syndrome was subsequently made. *Bartonella* serology and a conjunctival biopsy were taken, and he was started on oral doxycycline 100 mg twice daily. The left conjunctival granulomata, however, worsened despite the two-week course of oral doxycycline.

Conjunctival tissue culture from the biopsy grew *Sporothrix schenckii*. His *Bartonella* serology was negative. Thus, the diagnosis was revised to left ocular sporotrichosis, and oral itraconazole 200 mg daily was commenced. Marked improvement was seen at two weeks, and a complete resolution was achieved within three months of treatment. In total, he had completed 12 weeks of oral itraconazole without adverse effects. No recurrence was seen at one year of follow-up.

**Figure 3:**
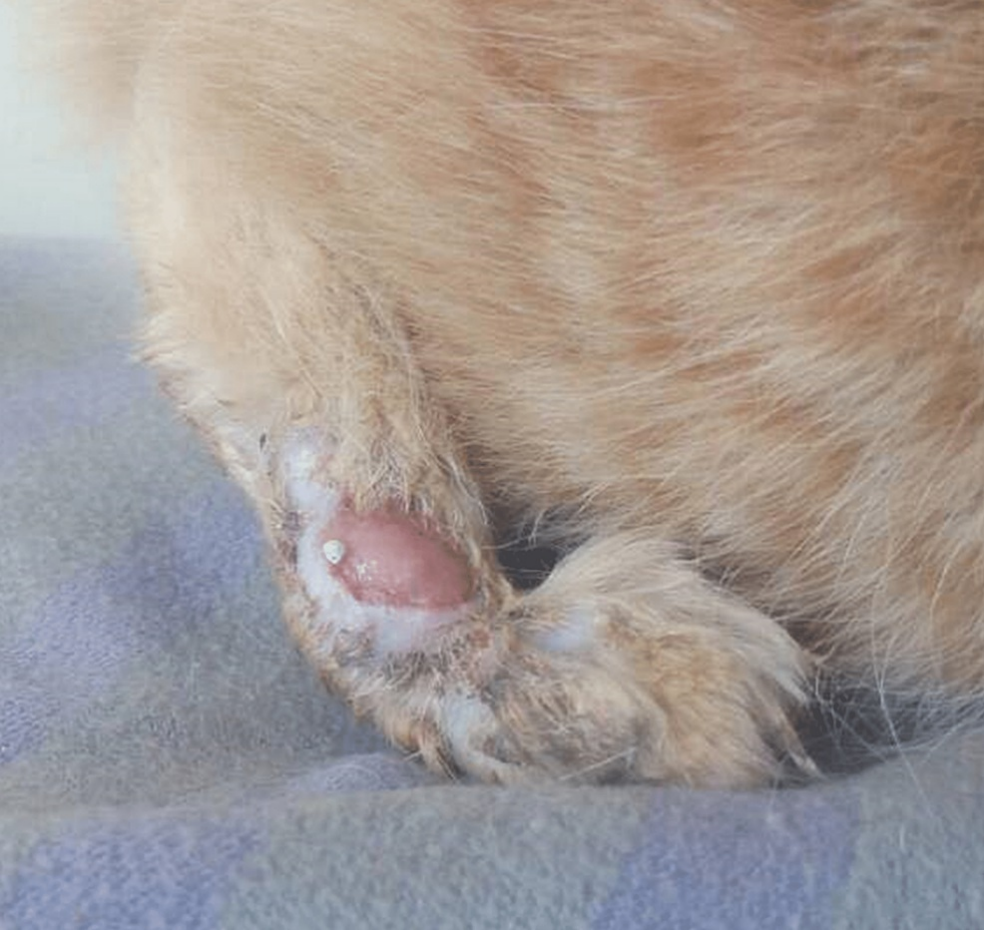
Skin lesion that was seen on the patient’s cat. The picture was used with permission from the patient.

We identified that the veterinarian treating the patient's cat had prescribed oral itraconazole for its "Sporo" cutaneous infection. Figure 3 shows the lesion that was seen on the patient's cat.

Case 2

**Figure 4:**
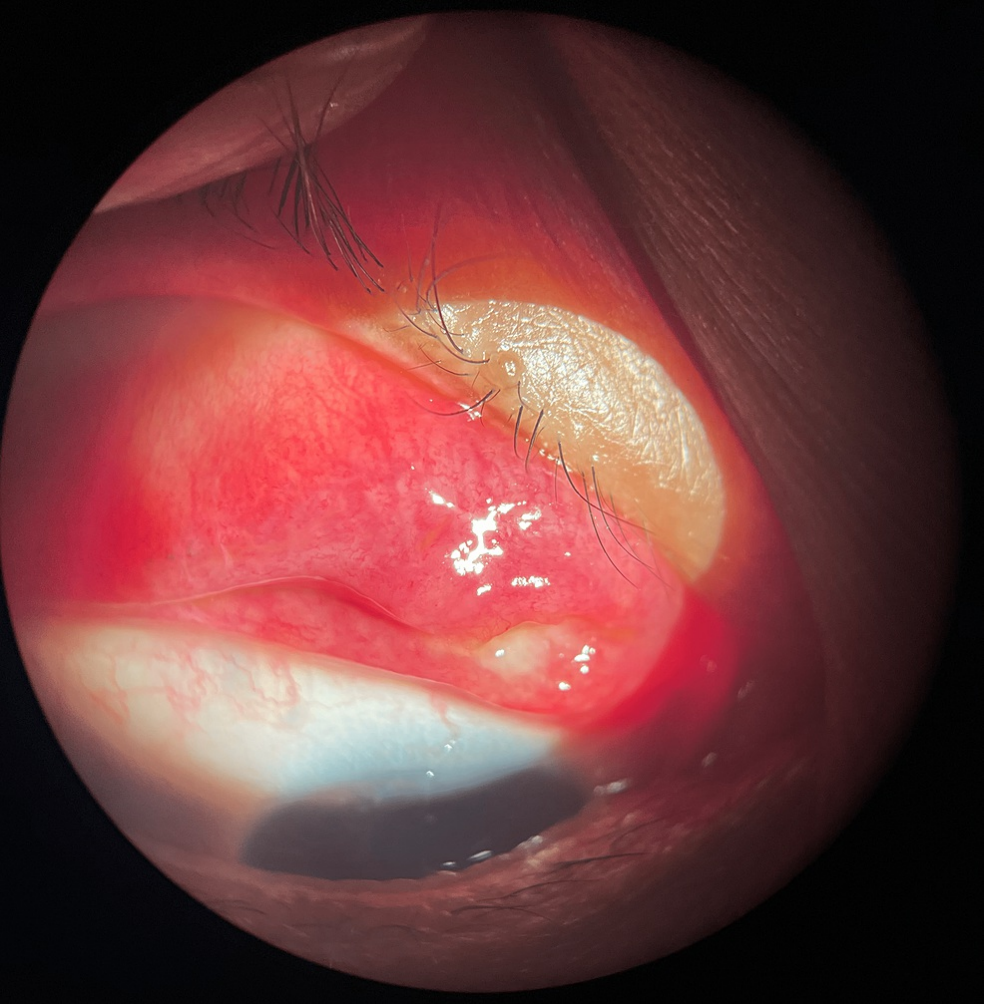
A bumpy upper eyelid tarsal conjunctival granulomatous lesion with yellowish discharge. A punctum was not seen.

A 32-year-old healthy lady was seen with a two-day history of right eye upper eyelid swelling, which was painless but caused some significant discomfort. She reported receiving a scratch on her wrist from her pet cat five days prior. This pet cat was also incidentally treated by a veterinarian with a medication labeled "for Sporo" for a cutaneous lesion around the cat's ears. The examination was notable for a lumpy upper tarsal conjunctiva granulomatous lesion with some yellowish discharge. Her vision was not affected. There were also a few non-tender enlarged lymph nodes at the preauricular and jugular group of lymph nodes. She had no other skin lesions elsewhere, and her fundus findings were unremarkable. Figure 4 shows a bumpy upper eyelid tarsal conjunctival granulomatous lesion with a yellowish discharge. A punctum was not seen.

Given the suggestive history, she was given oral itraconazole 200 mg daily. A swab culture of the discharge was taken, which returned a week later with the growth of *Sporothrix schenckii*. By 10 weeks of treatment, she achieved complete resolution of the granulomata and was continued on the same regime for an additional two weeks. At six months of follow-up, no recurrence of the lesion was seen.

## Discussion

The presence of granulomatous conjunctivitis as seen in the above cases greatly mimics oculoglandular syndrome, which is most commonly caused by* Bartonella* *spp*. A lack of a therapeutic response to oral doxycycline, and close contact with cats should warrant further investigation.

*Sporothrix schenckii* is a ubiquitous soil-dwelling fungus predicated in tropical and subtropical climes [1]. Sporotrichosis commonly occurs amongst agriculture workers, but it may also result from direct inoculation via scratches, pricks, or bites from zoonotic host carriers such as cats. In both of the cases above, the feline host had already shown signs of infection prior to transmission to the human patient.

The gold standard for diagnosing sporotrichosis is by isolation of *Sporothrix schenckii* from affected clinical samples, such as skin and mucosa biopsies or body fluids. Samples are further analyzed by culturing, histopathological examination under direct microscopic view, and serological tests such as enzyme-linked immunosorbent assay (ELISA) [1,2].

In immunocompetent individuals, sporotrichosis is usually limited to skin or mucocutaneous manifestations. Disseminated diseases, including osteoarticular, pulmonary, meningeal, and systemic involvement, are usually seen in immune deficiency states. These include patients with acquired immunodeficiency syndrome (AIDS), long-term steroid therapy, or those on immunosuppressive agents.

In rare occurrences of ocular sporotrichosis, the forms described are granulomatous uveitis, retinitis, choroiditis, and progression to endophthalmitis. Hematogenous spread is the likely route of infection in these intraocular forms and is again related to immune deficiency states [2-4].

Meanwhile, the ocular adnexal forms of sporotrichosis include eyelid or eyebrow cutaneous lesions, granulomatous conjunctivitis, such as in these cases, or involvement of the nasolacrimal apparatus causing dacryocystitis. Conjunctival forms represent 2.3% of all ocular sporotrichosis presentations [3,5].

Itraconazole is the treatment of choice for several forms of sporotrichosis and treatment is recommended to be continued a further two to four weeks after resolution of lesions, typically three to six months [6].

It is nearly impossible to determine the scale of feline epizootic sporotrichosis as reporting is generally not done nor required as a regulation. The additional risk of interspecies transmission to veterinarians and owners, prolonged treatment times, and high costs are considerations that limit the ability to eradicate it within the cat population [7,8].

## Conclusions

Ocular sporotrichosis is rare but treatable. It should be considered in granulomatous conjunctivitis that does not respond to antibiotics or if there was feline contact. The feline host may show cutaneous signs of infection prior to the inoculation of the human patient. A conjunctival biopsy or swab culture helps confirm the diagnosis of infection with *Sporothrix schenckii*. Oral itraconazole is given for 10 weeks and continued for a further two weeks after the complete resolution of lesions, effectively treating conjunctival lesions and preventing recurrence.
